# (Self-)Reflexion and training of professional skills in the context of “being a doctor” in the future – a qualitative analysis of medical students' experience in LET ME ... keep you real!

**DOI:** 10.3205/zma001340

**Published:** 2020-09-15

**Authors:** Laura Scheide, Daniel Teufel, Marjo Wijnen-Meijer, Pascal O. Berberat

**Affiliations:** 1 TUM Medical Education Center, München, Germany

**Keywords:** socialization, social identification, self concept, self reflexion, professional role, medical humanities, narrative medicine, professional competence, evaluating methods, teaching methods

## Abstract

**Objective: **This paper seeks to assess how medical students can be trained in medical studies seminars to examine their own professional role as doctors. The *LET ME ... keep you real!* university seminar was developed and conducted at the Technical University of Munich. In this context, the following questions will be addressed: How can we assess the contribution of a university seminar to a medical student’s own examination of being a doctor? And: What skills are developed in *LET ME ... keep you real!* that foster medical students’ (self-)reflexion?

**Methods: **The source data is statements made by medical students who took part in the *LET ME….keep you real!* seminar from 2016-2018. Student perspectives were analyzed after five focus group discussions with a total of 26 medical student participants and two individual interviews. Based on the interpretative paradigm and following the credo of a methodological exploration of medical students’ lifeworld, their specific learning experience as well as their ability for (self-)reflexion were mapped out.

**Results: **The research questions guiding the assessment of the seminar can be answered as follows: From the students’ perspective, the (self-)reflexion triggered and organized by *LET ME ... keep you real!* can be seen as rehearsing a meta-view. From the students’ standpoint, five skills can be identified that make this behavior possible:

1. questioning and doubting,
2. recognizing relevant perspectives,
3. classifying viewpoints,
4. maintaining communal exchanges and
5. deciding on a (different) position.

Situatively, these skills are often used in combination and challenge students on an intellectual, communicative and emotional level.

**Conclusion: **The ability to (self-)reflect should be more strongly integrated in university medical training by providing appropriate support, especially since it presents students with specific challenges to (self-)reflexively approach their own future as doctors. The skills mapped out here can be used as orientation to develop seminars on professional (self-)reflexive identity development for medical students.

## Zusammenfassung

**Zielsetzung: **Ziel des vorliegenden Beitrages ist es, zu evaluieren, wie medizinstudentische Auseinandersetzung mit der eigenen professionellen Arztrolle schon während des Medizinstudiums in einem Seminar trainiert werden kann. Hierzu werden folgende Fragen beantwortet: Wie ist der Beitrag eines universitären Seminares, wie das an der TU München entwickelte und durchgeführte *LET ME ... keep you real!*, zu einer medizinstudentischen Auseinandersetzung mit dem Arzt-Sein zu erfassen? Und: Welche Fähigkeiten werden aus Studierendensicht hier trainiert, um medizinstudentische Selbst-Reflexion zu praktizieren?

**Methodik: **Datengrundlage bilden die Aussagen von Medizinstudierenden, die an dem Seminar *LET ME ... keep you real!* von 2016 bis 2018 teilgenommen haben. Mit Hilfe von fünf Fokusgruppendiskussionen mit insgesamt 26 medizinstudentischen Teilnehmenden und zwei Einzelinterviews wurden Studierendenperspektiven analysiert. Anhand des interpretativen Paradigmas und unter dem Credo eines methodischen Entdeckens der medizinstudentischen Lebenswelt wurden die spezifische medizinstudentische Lernerfahrung sowie die Fähigkeiten zur (Selbst-)Reflexion herausgearbeitet.

**Ergebnisse: **Die auf die Evaluation des Seminares ausgerichteten Forschungsfragen lassen sich wie folgt beantworten: Aus Studierendenperspektive kann die, durch *LET ME ... keep you real!* evozierte und geordnete, (Selbst-)Reflexion als das Einüben eines Metablicks gesehen werden. Es lassen sich aus Studierendensicht fünf Fähigkeiten herausstellen, die diese Verhaltensweise ermöglichen:

1. Hinterfragen und Zweifeln,
2. relevante Perspektiven erkennen,
3. Sichtweise einordnen,
4. gemeinschaftlichen Austausch pflegen und
5. sich für eine (alternative) Position entscheiden.

Situativ kommen diese Fähigkeiten oft kombiniert zur Anwendung und fordern Studierende sowohl auf intellektueller und kommunikativer als auch auf emotionaler Ebene.

**Schlussfolgerung: **Die Fähigkeiten zur (Selbst-)Reflexion sollten durch geeignete Hilfestellungen stärker Eingang in die universitäre medizinische Lehre finden, vor allem, weil es Studierende vor spezifische Herausforderungen stellt, sich (selbst-)reflexiv zu ihrem zukünftigen eigenen Arzt-Sein und den Arztrollen zu verhalten. Die hier aus einer Medizinstudierendensicht herausgearbeiteten Fähigkeiten können als Orientierung genutzt werden, um Seminare zur medizindidaktisch angeleiteten professionellen (selbst-)reflexiven Identitätsentwicklung bei Medizinstudierenden zu entwickeln.

## Introduction: dealing with being a doctor

Reflecting on what it means to be a doctor is a desirable part of medical training. This includes coping with the basic roles of a doctor, as defined by the Royal College of Physicians and Surgeons of Canada (CanMEDS) [1], and learning the medical skills highlighted in the *Nationaler kompetenzbasierte Lernzielkatalog Medizin* (NKLM, National Competency-based Learning Objectives Checklist for Medicine) [2]. These are very helpful in coming to terms with the high demands placed on medical professionals. In this context, in addition to addressing the many health, ethical, professional, social and interactional challenges responsibly, personal self-knowledge, self-criticism and self-development and, above all, self-reflexion are explicitly identified in the NKLM as key to competent professional conduct [http://www.nklm.de]. Schön has already established and investigated the necessity of reflexive practice for professional practitioners [2]. Reflexion may thus be considered a valuable part of everyday medical practice. The attempt to examine one’s being a doctor can be undertaken during medical studies, at least in order to mentally prepare oneself for the medical role that will be assumed later. This is where the concrete situations in which experiences, thoughts and feelings in the exploration of what it means to be a doctor should be brought into focus as facilitative of the learning process of reflexion for future physicians. This becomes possible if moments of reflexion are considered as part of a professional doctor’s identity formation. Cruess et al. describe professional identity development as a social internalization process that begins during medical studies and should also be supported by teaching: “Understanding that students acquire their identity by playing a role over time until the details of that role become internalized can guide the nature of training interventions” [3]. 

Numerous studies have already emphasized the importance of reflexive practices and call for greater incorporation of reflexion in medical education. Uygur et al. have assimilated these studies and offer an overview of effective teaching methods for developing doctor’s reflexive skills. One finding of their review is that guidelines for reflexive writing tasks, as well as supportive feedback on written output, are effective methods of choice for teaching reflexion because they provide the clearest measurable evidence for it. However, they also concede that a consistent definition of the process of reflexion is lacking [4]. Even beyond the discourses on evidence and measurement of learning outcomes, the reflexive individual with their affective states, attitudes and developmental potential is already recognized in the medical training literature as a subject of investigation. Here, however, reference is also made to different epistemological approaches and the dominating effect of a focus on evaluation and evidence, which standardizes the discourse on medical training [5]. In a recently published study, two overarching themes are identified as important for medical students in dealing with their own professional identity: The reflexion on one’s own social status (being) and reflexion on one’s target status (becoming). The results of this study also clearly show that both self-observations (me-to-me reflexions) and observations of socio-cultural contexts (me-to-them reflexions), in which medical students develop a professional identity, are of major importance [6]. It is thus evident that reflexion can be understood and investigated in different ways depending on the epistemological discourse. Our article takes up the issue of possible medical-training support for medical students in connection with these diverse possibilities for reflexion within the framework of a university seminar. 

The *LET ME ... keep you real!* seminar, offered for the first time in 2016 at the Faculty of Medicine at the Technical University of Munich (TUM), invokes professional subjectivity in examining one’s being a doctor [7], [8]. With regard to the evaluation of the learning content and the formation of medical training theory, this article answers two questions: How can we assess the contribution of a university seminar to a medical student’s own examination of being a doctor? And: What skills are developed in *LET ME ... keep you real!* that foster medical students’ (self-)reflexion?

## Methods

The explicit topic of this article and the focus group discussions, which serve as the primary source of data, were part of ethnographic and evaluative research accompanying the LET ME program. This was in line with the interpretive paradigm and emphasized the subjective nature of understanding human experience and reality construction to determine the usefulness of the training content [9]. At that point, our research project was principally concerned with understanding the effects of a seminar such as *LET ME ... keep you real!* from the perspective of the students’ lifeworld. In our view, this was best achieved initially by means of the methodological exploration since this could be followed without theoretical constraints on the relevance of the participants in a field [10]. In practice, this meant simply collecting the opinions of medical student participants in the seminar. This also entailed listening to their learning experiences in their examination of their being a doctor and taking them seriously in their diversity. Designed as accompanying research, the procedure followed the style of qualitative evaluation research according to the principle of “responsiveness”, i.e., research and development did not act independently of each other, but were understood to be communicatively interdependent [11]. Our findings were thus formulated with regard to medical training considerations and the scientific study also reflected their medical training effects. 32 criteria for reporting qualitative studies, in the form of the consolidated criteria for reporting qualitative research (COREQ), were used to classify and present the research findings for this article [12]. 

### Medical training context and thematic focus

*LET ME ... keep you real!* is one of the offers on the LET ME program, which also includes sessions that focus on other aspects of being a doctor, along with a reading circle. One of the basic ideas of the LET ME program (short for *Lettered Medicine/Lettered Medical Education*) is that the subjective dimension of both patients and practitioners plays a decisive role in view of the professional and personal challenges of everyday clinical activities [13]. The courses on the LET ME program are based on theoretical approaches drawn from the medical-training currents in *Professional Identity Formation* [14], Critical Medical Humanities [15] and Narrative Medicine [16]. Against this background, *LET ME ... keep you real!*, as the two-part pilot project of LET ME, was successfully implemented for the first time in the winter semester of 2016/17 and repeated in the following semesters, in some cases slightly revised. In two units of four hours each, the two central methods in *Narrative Medicine – close reading* and *reflexive writing* – were deployed [16]. In concrete terms, close reading means that both literary texts and film excerpts were closely studied and discussed collectively. In the subsequent* reflective writing*, the students were then given writing tasks tailored to their individual needs to allow them to formulate their own positions on the topics of the text and film excerpts. They could then read them aloud to each other and discuss them together. In addition, it was also part of the overall design to keep both units open-ended in terms of expected outcomes, so that thematic divergences and digressions sometimes lasting several minutes could occur and be discussed as a group. Both seminars were led by a humanities moderator and a medical expert. With regard to the teaching roles, the aim was to engage the students on an equal footing and thus explicitly limit the role of the instructors to a minimum. Instead, the opinions of the students as well as the opinions of the moderator and the medical expert were regarded as equally important. The *LET ME ... keep you real!* seminar was initially offered to the students as an elective course in addition to the current curriculum. In principle, it was open to all students, regardless of which stage of their studies they were in, although participation was limited to 8-13 participants. From 2016-2018, a total of 54 medical students participated in *LET ME ... keep you real!.*

#### Reflexivity of the researchers

The research process was designed by a sociologist (LS), who carried out the data collection, analysed the material, and developed the relevant research questions. This was done in constant discussion with the developer of the seminar format (DT, cultural scientist, M.A.), the doctor or medical educator (POB, Prof. Dr. med.) involved in the development and implementation, student assistants and the teaching research group for medicine at TUM. The researcher had spent approximately one year working on the conception and implementation of qualitative evaluation research [9], [11] in various fields of teaching in medicine at the TUM and finally developed the research project within the framework of the LET ME program based on this experience. There were no conflicts of interest in her role as a researching sociologist and government employee in relation to the medical students of the TUM. The LET ME seminars were not gradet. Nor, therefore, was the sociologist indirectly involved in grading processes.

#### Participants in the focus groups

The source data for this work was mainly provided by focus group discussions, which were the third phase of the *LET ME ... keep you real!* seminar. In the first phase of the seminar, the participants were informed that focus groups would take place. For each* LET ME ... keep you real!* session, there were 1-2 focus groups of three to six people each, depending on the number of participants. Five focus groups with a total of 26 medical students and one interviewer each were organized for the period 2016-18. The length of the focus group discussions ranged from 1 h 30 min to 2 h 10 min. The youngest TUM participant in one of the focus groups was born in 1997, the oldest in 1980. At the time of the seminar, the participant with the least experience of medical studies by semesters was in the 5^th^ semester, the one with the most in the 13^th^ semester. One seminar unit did not produce a focus group because the students were not willing to participate. Instead, two individual interviews of 1 h 25 min and 1 h 17 min were conducted. Participation in the focus groups was voluntary.

#### Data collection

The first focus group met half a year after the first *LET ME ... keep you real!* seminar. This allowed first conclusions to be drawn about the impact of the seminar. On the one hand, we continued to choose the focus group method of data collection, but this time one week after the seminar as it gave us the opportunity to gain insight into the thoughts and feelings of the students with regard to the behaviors encountered in the seminar. In addition, the questions were intended to encourage the students to explicate background knowledge that was taken for granted. On the other hand, we hoped that the group size of three to six people would also encourage quieter seminar members, whose perspective had been somewhat neglected in the seminar, to join in the discussion. One week after the seminar, we had the impression that the students were still able to report in great detail about their experiences in the seminar. Last but not least, the focus groups were also perceived by the participating students as a useful supplement to the examination of their being a doctor and as a further opportunity for exchange. The researcher was the moderator here, but the doctor and the developer of the seminar were not present. The participants were assured that the opinions expressed would only be passed on in anonymized form to the developers and leaders of the program. 

Each focus group was initiated by a prompt from the interviewer, in order to focus not only on the *LET ME ... keep you real!* seminar but also and above all on experiences that occurred outside the seminar. The opening question in the guided discussion was: 

Have you ever thought about what it means to be a doctor? 

(Then addressing the individual participants:) Tell me about a concrete situation in which something related to this crossed your mind... 

Once the students had started to talk about their general experiences in dealing with their being a doctor, the connection to the seminar was established: 

What effect has the seminar had on you? 

This introduction was supplemented by in-depth questions to gather reflexive statements and deeper insights into the topic under investigation [17]. The students were thus instructed to describe and evaluate the *LET ME ... keep you real!* seminar, to articulate its perceived learning effects, and to discuss their opinions about the seminar. Transcripts were prepared on the basis of audio recordings, which served as source data. 

#### Data analysis

**Figure 1:**
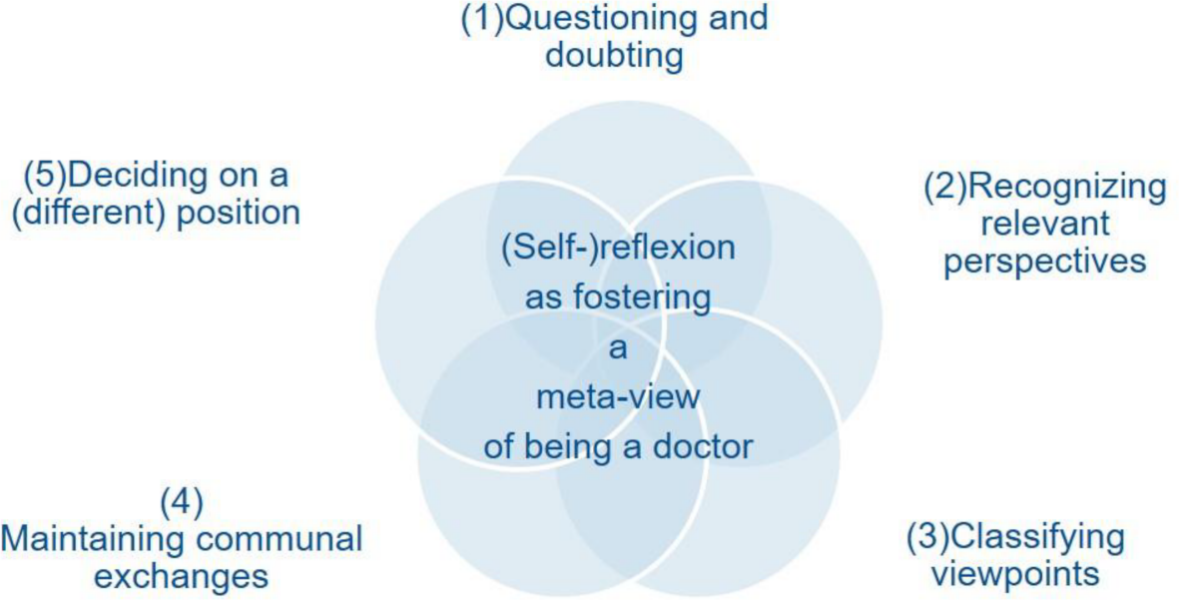
Student target profile for reflexion on being a doctor: (1)-(5) are the (self-)reflexive abilities which can be developed in the *LET ME… keep you real!* seminar for the purpose of practicing a situated meta-view of one’s being a doctor.

The formulation of the issue was specified step by step from the data material. The initial general research question was: How does one develop an understanding of being a doctor? And: How does one instigate this understanding? After a first overview of the relevant material in accordance with this general question, points relevant to medical training were selected. Furthermore, the relevant parts of the transcripts were open-coded by the researcher using MAXQDA 12 (VERBI GmbH, Berlin, Germany) software. This involves selecting, sorting, comparing and relating different units of meaning from the material according to our general research question by the same or different designations in order to abstract an analytical added value [10], [18]. The findings related to the research question in this paper were primarily developed using in vivo coding [19]. This means that care was taken to remain as close as possible to the formulations chosen by students. Subsequently, those passages were explored in greater depth in which the behavior that would be specifically cultivated by a seminar such as *LET ME ... keep you real!* from the students’ point of view was described (see the section: The contribution of the *LET ME ... keep you real!* seminar: Learning (self-)reflexion as a meta-view). From the nuanced descriptions and evaluations of numerous seminar situations, the various medical student abilities and their characteristics were abstracted whose combination seemed to produce the behavior triggered by the seminar context from the medical student’s perspective. In addition, the student evaluations and suggestions for improvement with regard to the seminar were included and analyzed. This enabled us to determine how individual seminar elements could be beneficial or detrimental to the learning of one or more of the above-mentioned skills from a medical student’s perspective (see the section: Training of student (self-)reflexive skills in *LET ME ... keep you real!*) With regard to the evaluation of the learning content and theory formation in teaching medicine, we have divided the general question into two parts: How can we assess the contribution of a university seminar to a medical student’s own examination of being a doctor? And: What skills are developed in *LET ME ... keep you real!* that foster medical students’ (self-)reflexion? We quote examples of data from the focus groups FG1 to FG5 or from the individual interviews EI1 and EI2, which all took place in the period from May 5/24/2017 to 5/7/2018. In addition, Attachment 1 shows our codes and their grouping. This serves as an overview and supplement to the text. Figure 1 (Fig. 1) shows a target profile, which shows the codes in their grouping as different levels of (self-)reflexion and may serve as an aide memoire.

#### Ethics

The students were made aware of the fact that the research focus is on understanding their perspectives and thus gaining an insight into the student lifeworld. The student participants were also assured of anonymity orally and in writing, and their written consent to participate in the research was recorded. The consent of the ethics committee at the TUM was sought and granted (495/18 pp.).

## Results: reflexion and self-reflexion on being a doctor from the medical student perspective

In the following subsections, we first give student descriptions of the seminar situation, which are the basis for our conclusions about the seminar’s contribution to examination of one’s being a doctor as a particular form of reflexion and self-reflexion. Subsequently, we present more in-depth student descriptions and evaluations, from which we abstract abilities for successful (self-)reflexion, which, from the point of view of the medical students could be developed in *LET ME ... keep your real!. *

### The contribution of the LET ME ... keep you real! seminar: Learning (self-)reflexion skills as a meta-view

The following example shows how (self-)reflexion in a university seminar – in the sense of a constructive switch between “personal” perspective and “abstract” (FG2) meta-perspective – can be successful.

Student: *I think that somehow this helps to pick out, collect and present arguments. [...] And that’s what you learn here [in the seminar, note L.S.] I think so, too. Just to express yourself, to read and present things, to accept opinions, to position yourself and other matters and then to find a compromise somewhere. [FG1]*

Together with other parts of the material, a skilful medical-student examination of being a doctor can be understood as a reflexive and self-reflexive ability. This (self-)reflexive ability makes it easier to recognize different perspectives on the medical world and thus to consciously enter into the process of examining one’s being a doctor. The seminar can be helpful in enabling participants to go “into depth” (FG4) with the help of a practiced meta-view or to gain an expanded view. 

Student: *You learn this meta-view, to step out of your own perspective and to look at the bigger picture. (FG1)*

Learning experiences in the seminar include, for example, discussions and own thoughts. To evaluate these experiences as a measurable performance is seen as a critical undertaking.

Student: *[...] we have to professionalize it a bit, so that we produce something, but in such a way that the format, which we all think is great, is somehow preserved. […]*

Interviewer: *What is your opinion on that, or, on the comment, that something has to come out of it, if I can summarize it that way?*

Student:* [...] there is something going on that you can’t measure. That’s the problem. And the problem with the whole thing. (FG1)*

(Self-)reflexion through the use of impulses for reflexion from the humanities and the arts, can be described as a “nice change” (FG3) and “stimulus” (FG3) from the perspective of medical students, but can also be perceived as “strange” (EI1). The intense discussion in the seminar can even be regarded as a “waste of time” (EI2).

Student: *Maybe as a criticism, but you can say that about any course, that there is a lot of talking, and often without much in the way of results. And we are so attuned to the fact that we have to learn a great deal in a very short time or have to absorb a great deal, and when you talk a lot without getting anywhere, that’s just a little bit/ sometimes you have the feeling that, okay, that was a bit of a waste of time. (EI2)*

For some medical students, however, the seminar context also shows how important and necessary it is for them to successfully deal with being a doctor themselves. This is then explained as a feeling of security.

Student: *[...] it is one of those questions that can stay with you, if you, yes, it can stay with you all your life, what is this profession in fact, why do I do it. [...] That’s why, yes, it gave me security, this seminar. I still don’t have such a clear picture, but that’s okay. (FG4)*

At its best, the seminar context reveals the “balancing act between medicine and society” (FG3) and the “broader fabric” into which medical professionals are woven (FG3), for example, through the “philosophical approach” (FG3) of the moderator or through the artistic written representation of the medical practitioner’s gaze in a haiku (FG3). 

A deeper understanding can also express itself in the recognition of the “multiplicity” (FG4) of being a doctor. Thus, elsewhere, an ambiguity is expressed regarding “the role of the doctor, or a role as a doctor perhaps sometime in the future, and what this means”, which was definitely “concretized” (FG4) in the seminar. To step out of one’s own perspective or to look at “the whole” (FG1) with the help of the meta-view can, depending on the task at hand, include a multitude of “meta levels of medicine” (FG5), e.g., one’s own ideals (FG1), historical developments (FG2), the balance between professional and private life (FG3), different conceptions of social roles (FG4), medical error culture (FG5), but also the doctor-patient relationship (EI1) and one’s own biography (EI2). 

#### Development of student (self-)reflexive skills in LET ME ... keep you real!

In the seminar, the constructive meta-view for practicing (self-)reflexion succeeds from a medical student perspective if at least one of five (self-)reflexive skills is the focus of the training. Certain seminar elements are presented by students as beneficial for the fostering of these skills, while others tend to inhibit it. 

#### Ability 1: questioning and doubting

Without creating an uncomfortable atmosphere for the students, the access to a free and meaningful doubt can be facilitated, as expressed in the following.

Student: *And that it [to think and to have doubts, L.S.] already has its meaning, especially if you question a lot for yourself and doubt a lot or something, which we all do, that it is also unsatisfactory or something, or you go round in circles. [FG1]*

Stepping out of the everyday medical student “comfort zone” (FG1) is described as an approach to the examination of one’s being a doctor.

Student: *There is no right or wrong in this seminar. There are opinions, there are feelings, there is no ‘this is now black and white, and this is what makes it so’. And this black and white, this thinking that you develop there, we never really looked at that in our regular studies [discussed, note: L.S.]. There are facts and there are the things you don’t know. (FG1)*

This stepping out of the comfort zone works particularly well for medical students when it becomes possible to question not only one’s own positions but also the perspectives of people who normally hold a position of power over the students. Abiding by the rule of equal status of all participants, students can be instructed to “free themselves a little bit from this socialization that takes place all the time [through medical studies, L.S. notes]” (FG4), which is also considered unsettling and difficult if their own motivation is lacking due to insufficient positive experiences (FG1). 

On top of this, it is reported that it is helpful to be able to address topics which “are still considered as unimportant” and where otherwise “it is not possible to say that something is really difficult to deal with” (FG4). Dealing with one’s own uncertainties (FG4) and weaknesses (FG2), medical decision errors (FG3), death of patients (FG5), or hierarchical thinking in medicine (FG1) are, for example, topics that from the students’ perspective are usually regarded as secondary in medical studies. 

#### Ability 2: recognizing relevant perspectives

Medical students also stress the importance of gaining access to other people’s opinions and experiences. In this way the “other view” (FG5) can be recognized. Relevant others can also be used as “role models” (FG2) or as negative examples that may leave one “quite shocked” (FG4), present “different models” of how to deal with others (FG4), “mirror” the effect on others (FG4), they can make you “realistic” again (FG4 in relation to the experienced doctor), give you access to a “generational view” (FG3 in relation to the experienced doctor), or show “how it works now, so to speak, for someone at a normal level” (EI1 in relation to the non-medical moderator). New insights are helpful for discussion, even outside the “small bubble” (EI2) in which some people see themselves in the everyday life of medical studies. 

Where possible, more heterogeneous discussion groups (e.g., students from all phases of study, experienced clinicians and non-physicians as moderators) are identified as helpful in guiding discussions from numerous perspectives by combining different perspectives, and even contradictory statements (FG4).

Student: *And I also found it interesting how similarly or how differently we saw things, and right at the end we formed an opinion about which qualities we find important as doctors. And then you could look back on it afterwards [...] which appeals to most people. (FG1)*

In particular, highlighting similarities and differences can be constructive in identifying relevant perspectives, as the quote illustrates. Ensuring this in a group size of 10-12 people can also be a problem.

Student: *In my opinion, a really intense conversation like this can only be conducted in pairs, so even with three people it becomes difficult, and with ten it’s a completely different ballgame. But especially when it’s about abstract or emotional issues, I don’t know, I think it helps to illuminate a topic from all sides, without judging it, maybe throwing all the aspects you can think of into the mix, but that’s just difficult when there are a lot of people. (FG4)*

Discussion in the group can also be associated with a certain “pressure to argue” (EI1), the mastery of which may appear to medical students to be worth striving for. 

#### Ability 3: classifying viewpoints

That it should be possible “to go into depth for oneself” (FG4) is mentioned. Participants saw it as helpful to want to and to be able to express oneself – orally and in writing – and to think through literary texts, film sequences, as well as suggestions from others regarding one’s own situation in life. A detailed and more precise making present of one’s self-perception, of “what is only vaguely apparent in one” (FG1), is definitely perceived as a beneficial outcome of the seminar. Open discussions in the group can support this “getting to the crux” (FG1). The process of making present is also explained as taking “a little distance” from oneself (FG1) or by asking oneself the questions, “Do I want this? Is this what I am?” (FG4). This can become problematic if the requisite time is lacking.

Student 1: *[...] and so you are showered with ideas from all sides and often don’t have time to go into depth for yourself regarding the input you just got because then the next one comes along. [FG4] *

As a guide to this ability, it is suggested that a facilitator can summarise and record different “views” on a topic from a “meta-position” and thus allow participants a “more tangible” idea of “where they would actually stand” (EI1). 

Student: *And then perhaps some of the differences that are now there may not be as stark as they seemed at first, or they may be just as stark, but then somehow you have an even clearer view of them (EI1). *

#### Ability 4: maintaining communal exchanges

The creation of a community that enables open discussions between peers so that “one is not alone with these fears or concerns” (FG4) is regarded as helpful. 

Student: *I really feel that I, I don’t really want to compare this, I think I have profited a lot from the experience reports, from [POB, note L.S], [...] where I had the feeling that it is in the room now, now we can talk about it. [...] That was somehow good, just for myself again, hey, you don’t have to be perfect. The others feel the same way. But it's about how you deal with it. And then in the end, I’ll have a much better idea of what I’m getting into. (FG2)*

To be allowed to cast one’s own experiences and feelings about being a doctor “into the room” (FG4) and to accept other opinions is regarded as important in order for the discussion to take place situatively. Furthermore, it is important to be able to “trust” one’s interlocutors (FG4), so that even on difficult topics one can express one’s own opinion. 

Another point made here is that in the context of a university seminar it is helpful to have participants “who wanted to be there” (FG1). In addition, according to the students, there is the danger of “steering the topic in another direction” or ceding the floor to someone else (FG4), e.g., if someone is perceived as an authority figure. The safe framework can then be endangered and communal feelings cannot develop. There is a discussion here about the extent to which an “intense conversation”, which evokes strong feelings, is possible at all in a group size like the one planned in the seminar (FG4). The evaluation of the discussions is also directed towards the consistent combination of different contributions and forced questioning. 

Student 1: *Of course there were situations where things were discussed more thoroughly, but otherwise I have the impression that someone said something and then maybe someone else said something about it, but there simply wasn’t the framework to really carry on elaborating thoughts or whatever, you know, to look at it, how do you really see things now [...] *

Student 2: *[...] maybe it’s only me who has an issue with it, but I often thought it a pity that this is the case.*

[…]

Student 2: *... was not used, what he [the experienced doctor, note L.S.] said, because I think it would have helped us. (FG4)*

#### Ability 5: deciding on a (different) position

The necessary return from the meta-perspective in order to draw one’s own conclusions can be a challenge both for the students and for the seminar design, as already pointed out in the student criticism, in which an unsuccessful discussion during the seminar is suspected of being a “waste of time” (EI2) (see Section 3.1). The desire for new positioning is also evident to varying degrees in the focus groups. At the most basic level, it can exhaust itself in formulating important questions. Some students describe the drawing out of new approaches as an impetus for change and personal development. This goes hand in hand with the feeling of being confronted with a fundamental “question”, which “every” person who is aware of the “demands that the job makes” (FG3) is confronted with. The confrontation with medical requirements is definitely evaluated as a positive feature of the seminar. 

Student: *But what I took with me, of course, everyone is confronted with the question, and it often came up during the seminar, how do I deal with the requirements of the job, how do I manage to lead a life worth living in addition to that, and what sacrifices am I willing to make in the course of my career. (FG3)*

In the most favourable case, however, a “new approach” can be generated through discussion in the seminar to initiate changes in the understanding of being a doctor and its moral consequences (FG1). An interaction between interviewees and a medical student during a focus group discussion illustrates the need to guide this skill through the seminar components. 

Interviewer: *But what for you personally is all of that now, where you would say, well, but we definitely have to perform that, or that’s where I have to make such and such a decision. That interests me. Is what I mean now a little clearer, or am I still not making myself clear? Then let’s try to get to the point together.*

Student 1: *Yes, not what the expectations of others are, but where I think I should do things that way, I should do this or that particular thing!*

Interviewer: *Right. So I’m simply interested in your personal perspective. Maybe different people get to different points. *

Student 2: *So I can imagine that each of us has the need to develop not only this analytical view [what is meant here is the medical-scientific view, which is introduced in the seminar as the doctor’s cold eye, note. L.S.], which is also important, but of course also to maintain a personal perspective and to do so in an appropriate way. Sure. So of course it is sometimes more important, so to speak, I don’t know, there are situations where empathy is more important, some where it is more important just to work through some kind of checklist. (FG3)*

What is triggered by the interviewer’s questioning of the student is the expression of the realization that constructive (self-)reflexive behavior includes the moment of decision-making. Another student subsequently expresses their assumption that medical training which supports an “appropriate way” (FG3) of changing perspectives might address a fundamental student need.

## Discussion: contribution of LET ME ... keep you real! to fostering reflexion on being a doctor

As per the specific research question underpinning this article, our method of analysis within the framework of the qualitatively oriented sociological evaluation research carried out at TUM MEC “takes into account the different relevance systems of researchers and research subjects in a systematic and controlled way”, without resorting to previously standardized categories [18]. 

With regard to the limitations of this work, especially with regard to the sampling strategy, the following should be noted: The student perspective elaborated here is only a selection from our research material and thus always a summary. Furthermore, it is, we assume, a necessarily abstract and idealized representation of a multitude of medical students’ needs for a constructive examination of one’s being a doctor. One reason for this is that so far only former participants in the *LET ME ... keep you real!* seminar have been chosen as participants in the focus groups. The data set, we infer, includes the opinions of a selection of students who are most likely predisposed to (self-)reflexion, also because participation in the seminar has so far been voluntary. This is certainly also reflected in the predominantly positive evaluation of the seminar. Another factor that should not be overlooked is the lack of willingness to participate in the seminar unit where the two individual interviews were conducted. Also, halo effect cannot be completely ruled out in the case of student criticism. 

Nevertheless, we would point out that in this initial study we do not claim to present a middle-range theory, which would be the aim of a comprehensive Grounded Theory, for example [19]. This was not possible at this time and with the data collected to date. Instead, we decided on the epistemological style of methodological exploration [10]. With regard to saturation for a comprehensive coverage of the student lifeworld in terms of content, it would certainly be important to interview further students, for example randomly selected non-participants, LET ME critics, or people who are about to begin their medical studies. Triangulation with other methods would also be helpful in subjecting seminar situations to closer observation and delving deeper into the individual perspectives of student attitudes, experiences and behavior. We consider the small number of participants – three to six per focus group – to be a particular strength of our data collection method and, moreover valuable, in terms of medical education. In this way, every participating student was able to have their say. In addition, a space was created in which students could express themselves without the presence of supposedly judgmental teachers. A focus group discussion can thus not only be used for data collection, but also offers additional moments of reflexion for the students and opportunities to learn from their own and others’ reflexions.

*LET ME ... keep you real!* contributes to the development of a professional medical identity, which from the students’ perspective can be understood as a meta-view that is guided by teaching theory in medicine. The inclusion of social contexts and a philosophical and artistic approach to thoughts, feelings and language provides a valuable approach to guiding a process of examining one’s being a doctor. A seminar such as *LET ME ... keep you real!* can, from the students’ point of view, promote the ability of medical students to(self-)reflect on being a doctor by providing them with mental training opportunities through which they learn to sensitise their own perception. Starting from the point of view of the medical students participating in *LET ME ... keep you real!* these (self-)reflexive abilities are recognizable if the descriptions and evaluations of the seminar are prepared analytically. The different abilities needed for (self-)reflexion in 1-5 often work in combination depending on the situation. If they are recognized in their variety and skilfully guided, they can at the very least be practiced within a university seminar. 

A seminar design can facilitate questioning if it absorbs the uncertainty associated with leaving the comfort zone of medical studies and provides space for articulated doubt. Elsewhere it has already been pointed out that a supportive learning environment is the prerequisite for reflexion to be possible for medical students [6], [20]. The students’ statements refer to an ability that is already being discussed in medical teaching discourse with reference to the keywords *Critical Medical Humanities* [15], [21], [22] and *critical thinking* [23]. The willingness to adopt an understanding and comparative stance, for example, to engage in narratives from the chosen stimuli for reflexion, but also to engage with the contributions of the other participants and thus to recognize and classify different perspectives, is repeatedly described by participants as essential. In the ideal case, as expressed by medical students, the perspectives worked out then become a multitude of newly discovered points of view, which students can then in turn reflect on and discuss openly in order to use them for their further development. From a medical student’s point of view, clarifying one’s own behavior, but also one’s feelings and ways of thinking in relation to one’s own experiences is an important part of the discussion. Reflective writing can help to make self-perception explicit [16]. *Close Reading* [24], [25], [26] can be supported, for example, by writing up different positions on the board and thereby illustrating different positions, which helps to situate one’s own position. Here it is also particularly important to provide ample time for reflexion. From the student’s perspective, constructive (self-)reflexion is also about adopting an attitude that makes examining being a doctor not only a personal matter but also a collective one. In concrete terms, this means that each individual student – with their attitudes, affective states and other characteristics and needs in relation to being a doctor – should be able to influence the course of a seminar geared towards (self-)reflexion, but is also jointly responsible for the depth of the discussion. Communal exchange is certainly a challenge that, according to some medical students, can also fail in the seminar if group members are not encouraged to offer contributions to the discussion. Unexpected arguments from discussions and taking up a position oneself are evaluated in the seminar as positive results and as an additional pool of valuable experience.

Inspiring stimuli for reflexion, the positions of an experienced medical professional, the impartial attitude of a moderator and the structured writing down of one’s own thoughts provide this support, because they not only address the experiences gained but also the emotional life of the medical students.

In respect of our considerations on the development of medical training theory, we maintain that medical training support of professional identity development [14] can start with a variety of doctor-related topics. It can aim to enable a constantly growing understanding of social role expectations – of the medical profession in general [1] – and a sensitized perception of being a doctor – for example, looking at the development of one’s own competencies [http://www.nklm.de]. This growth process, which is on the one hand intellectual, but on the other hand also emotional and communicative, is associated with challenges resulting from the perceived differences in status between the current status of a medical student (being) and the assumed target status of a competent medical practitioner (becoming) [6]. The student’s view, which is laid out in this article, enables a deeper understanding of a successful process: Dealing with medical challenges mentally is not only supported by addressing different medical issues, but also by reflexion on several levels. Each level of (self-)reflexion involves dealing with one’s own thoughts and feelings (me-to-me reflexions), but also processing the feelings and thoughts of others (me-to-them reflexions), as the study by Jarvis-Selinger et al. demonstrates [6]. The meta-view – as an extended professional medical perspective – is thus to be understood as the interplay of sensitized internal and external perception. It is to be understood as a way of being and seeing [5].

Guidelines for reflexion-stimulating writing tasks and supporting feedback on written documents are two options for standardizing reflexion activities so that the written explication of students’ internal and external perception of a medical issue can be measured [4]. In order to be able to carry out the different levels of (self-)reflexion with regard to being a doctor, however, a multitude of skills are required which are relevant across topics and which we doubt can be readily measured. With this understanding, we reject epistemological and evaluative frameworks that define grading assessments and the development of evidence in relation to reflexion as the only option [5]. The aim here is to advocate the use of the skills profile as an orientation, but not as rigid prescription for the development of seminars.

## Conclusions: studying one’s being a doctor (self-)reflexively

The mental examination of being a doctor is a crucial learning experience in medical-professional identity formation [3], brought into focus here as (self-)reflexion. It should therefore also be recognized and taught as a competence in the course of medical studies. If the competence of (self-)reflexion develops its full potential, it takes into account the expectations, recognized as important in the NKLM, of “responsible” and “independent” doctors who are capable of “integrating all medical roles” [http://www.nklm.de]. Seminars such as *LET ME ... keep you real!* are conducive to this competence, because it is about sharpening the medical students’ perception in order to acquire a meta-view: intellectually, communicatively, and also emotionally. The fact that broadening one’s perspective and looking at one’s own evaluations and feelings is a learning achievement should be explicitly emphasized by teachers. This is helpful in allaying the impression that mental examination of with being a doctor offers no added value and that the necessary skills for this are at best innate. Instead, space for joint thinking and the processing of experiences can probably be seen more as moments of emergence of valuable (self-)reflexive abilities. The skills identified here can serve as a target profile for initial orientation during training. The fact is, (self-)reflexion on being a doctor – like any medical competence – has to be practiced repeatedly. A meta-view that has been practiced during studies could also be conducive to the long-term reflexive behavior of medical professionals [2].

## Acknowledgements

Many thanks to René Schneider, M.A. and Philip Lambrix, M.A., for the cuts and corrections.

## Competing interests

The authors declare that they have no competing interests. 

## Supplementary Material

Tabelle 1: Auseinandersetzung mit dem eigenen Arzt-Sein im Rahmen eines universitären Seminars
