# Supplementary material for: (Self-)Reflexion and training of professional skills in the context of “being a doctor” in the future – a qualitative analysis of medical students' experience in LET ME ... keep you real!
Source: GMS J Med Educ. 2020 Sep 15;37(5):Doc47. doi: 10.3205/zma001340 (PMC7499461; doi:10.3205/zma001340)
Supplement: Tabelle 1: Auseinandersetzung mit dem eigenen Arzt-Sein im Rahmen eines universitären Seminars [file JME-37-47-s-001.pdf]

Table 1: *Dealing with one's being a doctor within the framework of a university seminar*

Student designations	Medical teaching focus for use in *LET ME…keep you real!*	
	Grouping of Codes as…	Codes
…the **attitude triggered** by the seminar (at least in-vivo)	Reflexive und self-reflexive earning process	"seeing the whole", "going into depth", "stepping out of one's own perspective", "philosophical approach," "creative written presentation", "dealing with questions that can stay with you your whole life", "switching between personal and abstract perspectives", "professionalism".
…of a **behavioral effect** (in-vivo)	practiced meta-view	"depth", "meta-view", "nice change", "stimulating", "alienating", "waste of time", "balancing act between medicine and society", "woven into a 'broad fabric'", "recognition of 'diversity'", "security", "finding a compromise".
...of a **desired thematic focus**	meta-levels of being a doctor	"meta-levels of medicine", own ideals, historical developments, balance between work and private life, different conceptions of social roles, medical error culture, doctor-patient relationship, own biography, "concretize role as doctor in the future"

Attachment 1 to: *Scheide L, Teufel D, Wijnen-Meijer M, Berberat PO. (Self-)Reflexion and training of professional skills in the context of "being a doctor" in the future – a qualitative analysis of medical students' experience in LET ME … keep your real!.* GMS J Med Educ. 2020;37(5):Doc47.
DOI: 10.3205/zma001340

	Medical teaching focus for use in *LET ME…keep you real!*	
Student designations	Grouping of Codes as…	Codes
…of a characteristic of a (self-)reflexive **ability** (In-vivo)	1 Questioning and doubting	Stepping out of your “comfort zone”,<br>“no black and white”, “opinions”,<br>“feelings”,<br>“a little bit of socialization”,<br>“unsettling”.
	2 Recognizing relevant perspectives	“different view”,<br>“role models”,<br>“shocking” negative examples,<br>“various models”,<br>“mirror”,<br>“realistic”,<br>“generation gap”,<br>effect on “normal level”,<br>outside the “little bubble”,<br>“from all sides”
	3 Classifying viewpoints	“a little distance” to yourself,<br>“Do I want that?”,<br>“Is that who I am?”,<br>feeling of “getting to the point”,<br>“vague” things,<br>“face up to uncertainties”.
	4 Mainaining communal exchanges	“not alone with fears and worries”,<br>“talk about it”,<br>“others feel the same”,<br>inspire “confidence”.
	5 Deciding on a (different) position	“new approach”,<br>morally “appropriate personal perspective”,<br>“appropriate way” of switching perspective,<br>awareness of the “fundamental question”.

Attachment 1 to: *Scheide L, Teufel D, Wijnen-Meijer M, Berberat PO. (Self-)Reflexion and training of professional skills in the context of “being a doctor” in the future – a qualitative analysis of medical students‘ experience in LET ME … keep your real!.* GMS J Med Educ. 2020;37(5):Doc47.
DOI: 10.3205/zma001340

Student designations	Medical teaching focus for use in *LET ME…keep you real!*	
	Grouping of Codes as…	Codes
…of guiding an ability in **the seminar** through	1 Questioning and doubting	Emphasize meaningfulness,<br>Allow dissatisfaction,<br>Discuss “incidentals”,<br>Discuss uncertainties,<br>Discuss weaknesses,<br>Discuss erroneous decisions,<br>Death of patients as a borderline situation
	2 Recognizing relevant perspectives	As heterogeneous as possible discussion group,<br>Formulate an opinion,<br>Highlighting similarities and differences,<br>Remain unbiased
	3 Classifying viewpoints	Moderator with summarizing “meta-position”,<br>Open discussions,<br>Plan individual times,<br>Oral expression,<br>Opportunity for written expression,<br>Creative input on own situation in life
	4 Maintaining communal exchanges	Cast experiences and sensations “into the room”,<br>Acceptance of other opinions,<br>Connecting contributions consistently,<br>Forced questions,<br>Voluntary participation
	5 Deciding on a (different) position	Formulate important questions,<br>Identify important situations and the “appropriate way” to (self-)reflect

Student designations	Medical teaching focus for use in *LET ME…keep you real!*

Attachment 1 to: *Scheide L, Teufel D, Wijnen-Meijer M, Berberat PO. (Self-)Reflexion and training of professional skills in the context of “being a doctor” in the future – a qualitative analysis of medical students‘ experience in LET ME … keep your real!.* GMS J Med Educ. 2020;37(5):Doc47.
DOI: 10.3205/zma001340

	Grouping of Codes as…	Codes
…of the **inhibiting** of training in the seminar through	1 Questioning and doubting	unresolved uncertainty, too little self-motivation, unpleasant atmosphere Hierarchical thinking
	2 Recognizing relevant perspectives	too big a group, unresolved “pressure to argue”
	3 Classifying viewpoints	time bottlenecks
	4 Maintaining communal exchanges	neglecting questions, avoiding topics, authorities
	5 Deciding on a (different) position	discussion without result

Note. Analytical steps of breaking up the data. Medical student perspective on the benefits of a seminar like *LET ME...keep you real!* with regard to the examination of one's being a doctor

Attachment 1 to: *Scheide L, Teufel D, Wijnen-Meijer M, Berberat PO. (Self-)Reflexion and training of professional skills in the context of “being a doctor” in the future – a qualitative analysis of medical students‘ experience in LET ME … keep your real!.* GMS J Med Educ. 2020;37(5):Doc47.
DOI: 10.3205/zma001340
